# Successful laparoscopic management of paraesophageal hiatal hernia with upside-down intrathoracic stomach: a case report

**DOI:** 10.1186/s13256-015-0519-6

**Published:** 2015-03-04

**Authors:** Sze Li Siow, Sze Chee Tee, Chee Ming Wong

**Affiliations:** 1 Department of Surgery, Jalan Hospital, 93586 Kuching, Sarawak Malaysia; 2 Department of Surgery, Faculty of Medicine and Health Sciences, Universiti Malaysia Sarawak, 94300 Kota Samarahan, Kuching, Sarawak Malaysia

**Keywords:** Laparoscopy, Upside-down stomach, Gastric volvulus, Hiatal hernia, Fundoplication

## Abstract

**Introduction:**

Paraesophageal hernia with intrathoracic mesentericoaxial type of gastric volvulus is a rare clinical entity. The rotation occurs because of the idiopathic relaxation of the gastric ligaments and ascent of the stomach adjacent to the oesophagus through the hiatus defect, while the gastroesophageal junction remains in the abdomen. The open approach remains the gold standard therapy for most patients. Here we report the case of a patient with such a condition who underwent a successful laparoscopic surgery. A literature search revealed that this is the first case report from Southeast Asia.

**Case presentation:**

A 55-year-old Chinese woman presented to us with symptoms suggestive of gastric outlet obstruction for one year. A chest radiograph showed an air bubble with air-fluid level in her left thoracic cavity, where a diaphragmatic hernia was initially suspected. A computed tomography scan and barium swallow study demonstrated the presence of a type III paraesophageal hernia with intrathoracic upside-down stomach. A laparoscopy was performed and the herniated stomach was successfully reduced into the abdomen. The mediastinal part of the hernial sac was excised. Adequate intraabdominal length of oesophagus was achieved after resection of the sac and circumferential oesophageal dissection. A lateral releasing incision was made adjacent to the right crus to facilitate crural closure. The diaphragmatic defect and the hiatal closure were covered with a composite mesh. A Toupet fundoplication was performed to recreate the antireflux valve. She had an uneventful recovery. She had no relapse of previous symptoms at her six-month follow-up assessment.

**Conclusions:**

Laparoscopic repair of such a condition can be accomplished successfully and safely when it is performed with meticulous attention to the details of the surgical technique.

## Introduction

Gastric volvulus is classified into three types according to the axis of rotation: organoaxial (rotation around the long axis connecting the cardia and the pylorus); mesentericoaxial (rotation around the short axis connecting the lesser and greater curvatures) and combined (rotation around both the short and long axis). The mesentericoaxial type, as in our case, is a less common variant, occurring in 29% of cases [1]. The combination of a mesentericoaxial type of gastric volvulus and an intrathoracic location of the stomach is a rare clinical entity. Even though sporadic cases of intrathoracic gastric volvulus associated with paraesophageal hernia (PEH) have been reported in Europe and the United States, it is very rare in Southeast Asia. To the best of our knowledge, this is the first report from this region documenting this rare type of gastric volvulus entity with successful treatment using a laparoscopic approach.

## Case presentation

**Figure 1:**
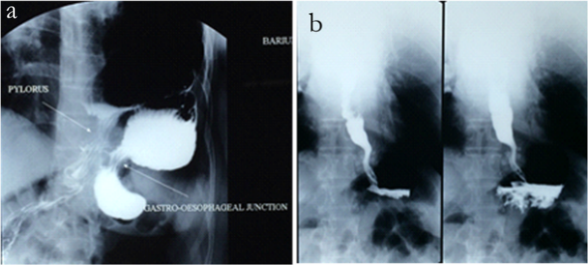
**Barium swallow study showing the upside-down appearance of the stomach in the thorax with the subdiaphragmatic location of the gastroesophageal junction before surgery (a) and normal subdiaphragmatic position of the stomach one month after the surgery (b).**

A 55-year-old Chinese woman presented with a history of left hypochondrial pain for a duration of one year, associated with early satiety and postprandial vomiting. She did not report any reflux symptoms. Her physical examination did not reveal any significant abnormality. An upper endoscopy did not show any evidence of reflux oesophagitis, except for a severely deformed stomach with difficult duodenal intubation. However, a chest radiograph showed an air bubble with air-fluid level in her left thoracic cavity, and a diaphragmatic hernia was initially suspected. A computed tomography (CT) scan of her abdomen and a barium swallow study (Figure 1a) confirmed a diagnosis of PEH with intrathoracic mesentericoaxial gastric volvulus.

**Figure 2:**
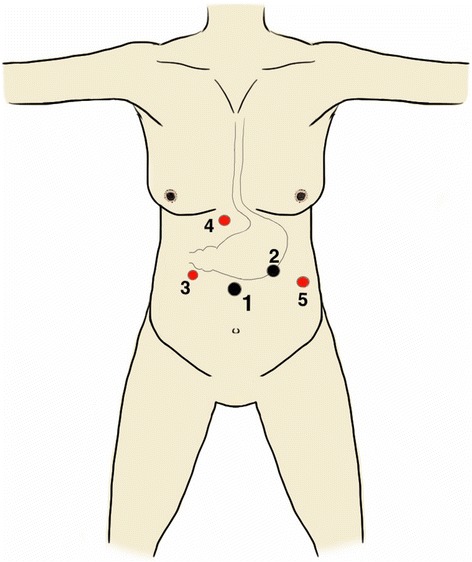
**Port position.** 1: Supraumbilical 12-mm camera port; 2: Left mid-clavicular 11-mm right-hand working port; 3: Right mid-clavicular 5-mm left-hand working port; 4: Subxiphoid 5-mm liver retraction port and 5: Left anterior axillary 5-mm retraction port. (Black denotes 11 to 12 mm ports and red denotes 5mm ports).

The surgery was performed with her in a modified lithotomy position, under general anaesthesia. The surgeon stood between her legs (the French position), with the camera surgeon at her right side and the assistant at her left side. Five trocars were used (Figure 2): one supraumbilical 12mm camera port, one 11mm left midclavicular right-hand working port, one 5mm right midclavicular left-hand working port, one 5mm left anterior axillary retraction port and one 5mm subxiphoid Nathanson liver retractor port (Cook Medical, Bloomington, USA). The initial entry into the abdomen was obtained with a bladeless 12mm trocar (XCEL®, Ethicon Endo-surgery, Cincinnati, USA) under direct telescopic visualization using a 10mm 0° laparoscope (Karl Storz Endoscopy, Tuttlingen, Germany). Once all the trocars were inserted, she was tilted into the reverse Trendelenburg position (20 to 30°).

**Figure 3:**
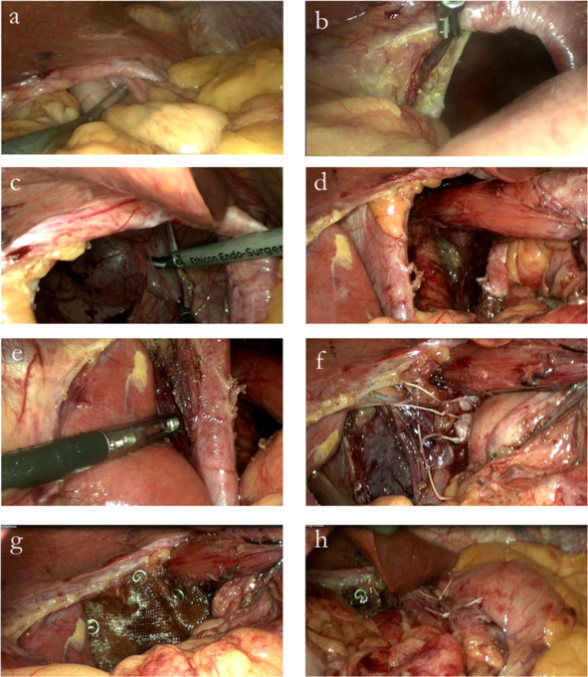
**Operative steps. a)** Reduction of the stomach from the thorax into the abdomen; **b)** Resection of the hernial sac started below the hiatal rim with division of the phreno-oesophageal ligament to correctly identify the plane between the mediastinal sac and the right crus; **c)** Dissecting the sac from the mediastinum; **d)** The wide hiatus visualized after dissection; **e)** Lateral releasing incision made adjacent to the right crus; **f)** Posterior approximation of the crura using Ethibond™ 2/0 sutures; **g)** Overlying of the composite mesh over the diaphragmatic defect and crus, secured with tacks; **h)** Formation of the Toupet fundoplication.

Initially, the herniated stomach was reduced using atraumatic graspers (Johan, Karl Storz Endoscopy, Tuttlingen, Germany) (Figure 3a). Gentle ‘hand-over-hand’ traction was applied to the stomach until complete reduction was achieved. Next, dissection of the hernia sac started below the hiatal rim. Division of the attenuated phreno-oesophageal ligament where it attached to the hiatal rim was made, followed by correct identification of the plane between the right crus and the hernia sac (Figure 3b). The sac was dissected and separated from the mediastinum, using a combination of sharp and blunt dissection (Figure 3c). The separation was assisted by pulling the cut edge of the sac into abdomen. Complete circumferential dissection of the sac as a whole was done in two parts, first the anterior and lateral sac in one piece, followed by the posterior sac. When performing retro-oesophageal dissection, care was taken to preserve the posterior vagus nerve. A nylon tape was then passed behind the oesophagus at the level of the oesophagogastric junction to sling the oesophagus upwards to allow further circumferential dissection of the oesophagus. In addition, the use of a sling prevented direct grasping of the oesophagus for retraction, thus avoiding possible injury to it. The dissection was completed when the mediastinal sac had been retracted down beyond the lower oesophagus and over the proximal stomach.

The redundant portion of the sac was excised with an ultrasonic scalpel (Harmonic®, Ethicon, Cincinnati, USA) taking care to avoid injury to the oesophageal and stomach wall. A wide hiatus was visualized after the dissection (Figure 3d), making it impossible to approximate the crura. A lateral releasing incision adjacent to the right crus was subsequently made with an ultrasonic shear (Harmonic®, Ethicon, Cincinnati, USA) (Figure 3e), allowing primary closure of the hiatus posterior to the oesophagus with three interrupted Ethibond™ 2/0 sutures (Ethicon, Cincinnati, USA) using intracorporeal knots (Figure 3f). A composite mesh (Proceed®, Ethicon, Johnson & Johnson Medical, Norderstedt, Germany) was later placed over the diaphragmatic defect and the posterior crural closure, and secured by tack fixation (Figure 3g). A Toupet (posterior 270°) fundoplication was performed to recreate the anti-reflux valve (Figure 3h). The posterior fundus was first fixed to the right crus using Ethibond™ 2/0 sutures, followed by four more similar sutures between the fundus and each side of the oesophagus.

Postoperatively, she was started on oral liquids on the evening of the surgery and she rapidly progressed to a soft diet, remaining on this diet for four weeks. She was discharged on postoperative day one. A barium swallow study at one month after surgery showed a normally located stomach (Figure 1b). At a follow-up review of six months, she was well, with no relapse of symptoms.

## Discussion

PEH is an uncommon condition, with a reported incidence of 5% out of all hiatal hernias. It is classified as one of three types: type II (true PEH), type III (a mixed paraesophageal and sliding hernia) or type IV (contains viscera other than the stomach). Approximately 90% are type III and the other 10% are type II or IV [2]. Type III PEH, as in our case report, occurs more commonly in elderly and probably evolves from type I as a result of hernial enlargement [3]. Nevertheless, the natural history of PEH is poorly understood. Gastric volvulus is a known complication of PEH. The finding of an intrathoracic upside-down stomach in our patient is due to the ascent of stomach adjacent to the oesophagus and the idiopathic relaxation of gastric ligaments, while the gastroesophageal junction remained in the abdomen.

Our patient’s diagnosis was suspected from the finding of a retrocardiac air bubble with or without air-fluid level on the lateral view of a chest radiograph. The differential diagnoses to be considered included diaphragmatic and PEH. A CT scan of the chest and abdomen can provide information on the type of hernia. A barium swallow test can confirm the type of PEH, and provides information on rotation of the stomach, as in our case. An upper endoscopy is mandatory to exclude the presence of oesophagitis or Barrett’s oesophagus that may have resulted in a shortened oesophagus. However, the procedure may be complicated by difficult intubation of the duodenum due to the malrotation of the stomach.

Elective repair is generally indicated for symptomatic PEH in otherwise fit patients with large hiatal hernias [4,5]. It is the preferred option as emergency surgery is associated with a higher rate of morbidity and mortality, as demonstrated in a population-based study [4]. However, in asymptomatic or minimally symptomatic PEH, the traditional rationale of prophylactically repairing all PEH in order to prevent life-threatening complications has been challenged in recent years, with the suggestion that this group of patients could be managed non-operatively [5]. Stylopoulos *et al.*, in a pooled analysis of several studies, estimated that the annual risk of PEH developing acute symptoms requiring surgery in such group of patients is 1.1% [5]. They also highlighted the overestimation of the mortality rate associated with emergency surgery, previously reported to be more than 40%, to be 5.4%.

The management of PEH has evolved over the last decade, from open thoracic to open abdominal and finally laparoscopic transabdominal approaches. The paradigm shift in treatment is attributed to the development and refinement of laparoscopic techniques for anti-reflux surgery and improvement in modern laparoscopic equipment and energy devices. Since the first description of the laparoscopic approach for large PEH in 1992 [6], it has become apparent that the laparoscopic approach is associated with significantly lower perioperative morbidity and shortened convalescence when compared to laparotomy or thoracotomy.

The key issues surrounding the laparoscopic repair of PEH are: (1) shortened oesophagus, (2) the need to excise the mediastinal hernia sac, (3) the method of crural closure and (4) the need to perform a gastropexy.

Shortened oesophagus is a real and important clinical entity in patients with PEH, with a reported incidence of 11% [7]. It is thought to arise as a result of either chronic inflammation or anatomic changes associated with herniation [7]. Careful preoperative endoscopic evaluation for the presence of either circumferential oesophagitis or Barrett’s oesophagus is important, as they may indicate the degree of perioesophageal inflammation, and possibly loss of oesophageal compliance and length. The final diagnosis of shortened oesophagus is always made in the operating theatre after adequate circumferential oesophageal mobilization. The failure to gain an adequate intraabdominal length of the oesophagus mandates an oesophageal lengthening procedure, such as a Collis gastroplasty, which should only be used sparingly or when absolute necessarily, as the procedure is associated with morbidity related to gastric resection and stapling [7]. Further, it results in the retention of acid-producing parietal cells in the neo-oesophagus above the intact fundoplication [7]. Nevertheless, most authors agree with the fact that the required length of the intraabdominal oesophagus is nearly always attainable with a complete resection of the mediastinal sac and circumferential oesophageal mobilization [7,8].

The resection of mediastinal sac achieves several other objectives. Firstly, it allows for the descent of the oesophagus into its normal position. Secondly, it eliminates the serous membrane lining the cavity of the mediastinum, thus reducing the risk of seroma. Thirdly, it eliminates the traction on the stomach that may cause recurrence of intrathoracic hernia [9]. Our patient did not have reflux symptoms nor was there any evidence of oesophagitis or Barrett’s oesophagus on an endoscopic assessment. We were able to achieve an intraabdominal oesophageal length of 3cm after adopting the above-mentioned technique, with the aid of nylon tape at the gastroesophageal junction. The redundant sac over the upper stomach was resected to facilitate identification of the anterior oesophagus and gastro-oesophageal junction.

The use of meshes in hiatal repair is controversial because of the concern of mesh-related complications. A mesh is used to reinforce the crural repair because it has been observed that failure of the crural closure, occurring in 5.7 to 11% of patients, is the main reason for postoperative intrathoracic migration of the Nissen fundic wrap following laparoscopic antireflux surgery [10]. The risk of hernial recurrence is proportionate to the size of the hiatal defect [11]. Generally, a reinforced hiatoplasty is recommended for patients with a hiatal size of more than 5cm^2^ to decrease the risk of hernial recurrence [11]. Two prospective randomized trials have demonstrated a significant reduction of recurrence when using mesh for crural closure [10,12]. Another study observed that a change in practice from primary crural repair in Nissen fundoplication to tension-free hiatoplasty using a polypropylene mesh led to a zero incidence of postoperative wrap herniation, in contrast to an earlier incidence of 13.8% [13].

While there are concerns of mesh-related complications, most authors did not report such untoward complications [10,12,13]. One consideration for mesh reinforcement is the placement of the mesh, which should not be in contact with the posterior aspect of the oesophagus, but with the posterior fundic wrap [10,13]. Other important considerations for mesh reinforcement of the hiatal closure are that: (1) the inert muscular properties of hiatal fibres that are poor in satellite cells and extracellular content, important in the cicatrization process, leads to a weakness in the scar tissue [13] and (2) change in direction of tension during inspiration, normally directed toward the vertebral insertion of the crura in normal circumstances, to retraction of the muscle fibres of the hiatus when the crura are sutured together [13]. In our patient, laparoscopic closure of the crura was not possible as both the crura were wide apart. We were left with the option of either patching the enlarged hiatus without crural approximation (leaving the passage for the oesophagus) in a true tension-free repair, or to perform a relaxing incision over the right crus to facilitate primary closure of the hiatus, followed by the use of on-lay mesh to cover the defect or to buttress the crural repair. The concept of tension-free repair using mesh is the most ideal approach in any hernial repair. We were concerned about the rupture of the mesh and recurrent herniation if our patient were to have excessive coughing or retching during the immediate postoperative period. Therefore, we performed a lateral releasing longitudinal incision in the diaphragm adjacent to the right crus in order to achieve crural approximation. The Proceed® composite mesh used in this case is made of polypropylene on one side and oxidized regenerated cellulose layer on the other side which minimizes bowel adhesions. The rationale of using a mesh in our case was to reinforce the diaphragmatic defect and the crural closure, as there was concern for disruption since both crura had been approximated under tension.

An antireflux procedure was performed as part of the repair as it has been shown that most patients with a PEH have evidence of reflux on pH monitoring [14], and failure to perform an antireflux procedure has been shown to result in postoperative reflux in 20% of patients [3]. In addition, patients without preoperative reflux may develop postoperative reflux, attributable to dissection and mobilization of the gastro-oesophageal junction [15]. Our patient had no symptoms of reflux, most probably attributed to the presence of a distorted gastroesophageal junction of the intrathoracic upside-down stomach. A Toupet fundoplication was performed as a partial wrap was the procedure of choice for laparoscopic antireflux surgery in our department. The interposition of the wrap between the mesh and the posterior oesophageal wall prevents direct contact of the oesophagus against the mesh. The evidence to support the ideal type of fundoplication is lacking, with both partial (Toupet or Dor) and complete (Nissen) wrap being described. In general terms, the main objectives of a fundoplication are to anchor the stomach at its subdiaphragmatic position and to effectively control the gastroesophageal reflux disease [7].

The role of an anterior gastropexy to prevent intraabdominal gastric volvulus remains controversial. In our case, we did not perform gastropexy, as the Toupet fundoplication with its wrap anchored to the oesophagus and right crus will inevitably prevent any recurrent volvulus.

## Conclusions

Our case highlights a successful laparoscopic approach to PEH with intrathoracic gastric volvulus and documents the use of a lateral releasing incision to facilitate crural closure and the placement of a mesh to reinforce a diaphragmatic defect and hiatal closure. However, the routine use of these two techniques as part of a PEH repair need to be examined in a large prospective randomized study with a long-term follow-up period.

## Consent

Written informed consent was obtained from the patient for publication of this case report and accompanying images. A copy of the written consent is available for review by the Editor-in-Chief of this journal.

## Abbreviations

PEH: Paraesophageal hernia
CT: Computed tomography
